# The Physician's Visiting List, 1888

**Published:** 1888-02

**Authors:** 


THE PHYSICIAN'S VISITING LIST. 1888. Lindsay and and Blakiston. Thirty-seventh year of its publication.

This concise visiting list appears for the present year in its familiar style, with much new matter added, but in such a manner as to maintain its usual size. This work answers for a dental appointment book, and is used for such a purpose by many dentists who appreciate its arrangement and durability. P. Blakiston, Son & Co., Philadelphia.

